# Subchronic Tolerance Trials of Graded Oral Supplementation with Phenylalanine or Serine in Healthy Adults

**DOI:** 10.3390/nu13061976

**Published:** 2021-06-08

**Authors:** Naoki Miura, Hideki Matsumoto, Luc Cynober, Patrick J. Stover, Rajavel Elango, Motoni Kadowaki, Dennis M. Bier, Miro Smriga

**Affiliations:** 1 Miura Medical Clinic, Higashitenma, Osaka 530-0044, Japan; nakano@clinical-rs.com; 2 Japan Branch of International Council for Amino Acid Science (ICAAS), Hatchobori, Tokyo 104-032, Japan; icaasjapan@icaas-npo.org; 3 Clinical Chemistry Lab., Cochin Hospital, Paris and Biological Nutrition Lab. and EA 4466, Faculty of Pharmacy, Université de Paris, 75014 Paris, France; luc.cynober@aphp.fr; 4 Scientific Advisory Committee of the International Council for Amino Acid Science (ICAAS), Avenue de Tervueren, 1150 Brussels, Belgium; Patrick.Stover@ag.tamu.edu (P.J.S.); relango@bcchr.ubc.ca (R.E.); kadowaki@niit.ac.jp (M.K.); dbier@bcm.edu (D.M.B.); 5 Texas A & M AgriLife, College Station, TX 77843, USA; 6 School of Population and Public Health, University of British Columbia, Vancouver, BC V6T 1Z4, Canada; 7 Department of Engineering, Niigata Institute of Technology, Niigata 950-0932, Japan; 8 USDA/ARS Children’s Nutrition Research Center, Baylor College of Medicine, Houston, TX 77030, USA; 9 International Council for Amino Acid Science (ICAAS), Avenue de Tervueren, 1150 Brussels, Belgium

**Keywords:** phenylalanine, serine, NOAEL, human, safety, dietary supplements

## Abstract

Phenylalanine and serine are amino acids used in dietary supplements and nutritional products consumed by healthy consumers; however, the safe level of phenylalanine or serine supplementation is unknown. The objective of this study was to conduct two 4-week clinical trials to evaluate the safety and tolerability of graded dosages of oral phenylalanine and oral serine. Healthy male adults (*n* = 60, 38.2 ± 1.8y) completed graded dosages of either phenylalanine or serine supplement (3, 6, 9 and 12 g/d) for 4 weeks with 2-week wash-out periods in between. Primary outcomes included vitals, a broad spectrum of circulating biochemical analytes, body weight, sleep quality and mental self-assessment. At low dosages, minor changes in serum electrolytes and plasma non-essential amino acids glutamine and aspartic acid concentrations were observed. Serine increased its plasma concentrations at high supplemental dosages (9 and 12 g/day), and phenylalanine increased plasma tyrosine concentrations at 12 g/day, but those changes were not considered toxicologically relevant. No other changes in measured parameters were observed, and study subjects tolerated 4-week-long oral supplementation of phenylalanine or serine without treatment-related adverse events. A clinical, no-observed-adverse-effect-level (NOAEL) of phenylalanine and serine supplementation in healthy adult males was determined to be 12 g/day.

## 1. Introduction

L-Phenylalanine (phenylalanine) and L-serine (serine) are amino acids ingested from regular foods, dietary supplements and products for special nutritional purposes. The mean daily intake of phenylalanine and serine from food sources was estimated at 3.4 g/day and 3.5 g/day, respectively, (all life stages and sex groups) based on the NHANES III [1]. Similar assessments of dietary intake were reported in the UK (mean intake of phenylalanine and serine each at 3.6–3.7 g/day) and Japan (mean intake of phenylalanine and serine each at 3.3–3.4 g/day) [2,3]. Given the confidence intervals around self-reported dietary intakes and given the ranges of nutrient contents found in food composition tables, intakes measured by these three national surveys are likely identical.

The NIH Dietary Supplement Label Database [4] records 101 dietary supplement products with serine and 40 products with phenylalanine. Most of these products are available online globally, and they illustrate an interest in the assumed benefits of both amino acids at levels substantially above the food intakes. Indeed, serine is a glucogenic amino acid and an important metabolic precursor in the synthesis of sphingolipids, other amino acids, including glycine and nucleotides [5]. Phenylalanine is a precursor of tyrosine and catecholaminergic neurotransmitters [6]. Recent peer-reviewed research with supplemental serine includes nutritional support of skeletal muscle regeneration [7] and treatment of amyotrophic lateral sclerosis [8]. Phenylalanine, being one of the nine essential amino acids, is regularly added to essential amino acid mixes that are used in an array of products, ranging from sport supplements intended for athletes [9] to foods and/or supplements addressing sarcopenia in elderly persons [10]. However, discussing the efficacy of ingesting the two amino acids as dietary supplements or functional foods is beyond the scope of this paper.

In view of the nutritional uses of both phenylalanine and serine and the lack of clinical safety data, the primary objective of this study was to determine the clinical no-observed-adverse-effect-level (NOAEL) for the subchronic (4 weeks) intake of supplemental phenylalanine and serine at 3–12 g/day and to add to the currently available literature on amino acid safety in human nutrition [11,12,13].

The experimental methodology was closely adopted from previous safety studies with other amino acids [14,15]. Due to the limited data available on biomarkers for clinical toxicity of either phenylalanine or serine, changes in a broad range of circulating biochemical analyte concentrations, anthropometry, macronutrient and caloric intakes, sleep quality, mental fatigue and any treatment-specific adverse effects were used as indicators of tolerance to increasing doses of both studied amino acids starting at the supplemental dose of 3 g/day.

## 2. Materials and Methods

The procedures in the two clinical trials were in accordance with ethical standards and were approved by the Miura Medical Clinic Ethics Review Committee (ethical approval code R1913 of 20 February 2020). The trials were registered in the clinical trial registration system operated by the University Hospital Medical Information Network Research Center under UMIN 000,040,281 (3 May 2020). The methodology was based on preceding clinical studies to determine the NOAEL of amino acids [14,15]. Subject recruitment, blood collection and anthropometric and body composition measurements were conducted in the Miura Medical Clinic and managed by a project coordinator with the proper training (Oneness Support Ltd., Osaka, Japan). The subjects provided written, informed consent before participation.

### 2.1. Subjects

The study population consisted of 60 healthy male subjects (age, 38.2 ± 1.8 years, height 173.5 ± 0.9 cm, weight 70.9 ± 1.5 kg). Participants were publicly recruited in the Osaka city area (Japan) and divided into two separate groups of 30 subjects, one receiving graded doses of phenylalanine and the other receiving graded doses of serine. During screening, all participants completed a health history and physical activity questionnaire that included current and recent medications and supplement use. Inclusion criteria were as follows: free from chronic disease as determined by the medical staff; BMI ≥ 18 and ≤ 27 kg/m^2^; baseline blood pressure < 140/90 mm Hg; and able to swallow multiple capsules.

Exclusion criteria were as follows: phenylketonuria, known musculoskeletal disease (e.g., rheumatoid arthritis), enteropathy, respiratory or cardiac disease, a movement disorder that might affect skeletal muscle mass, function or metabolism (e.g., diabetes mellitus); long-term treatment with exogenous hormones or other pharmacological interventions; excessive alcohol intake; and any other condition according to the appointed medical staff that would interfere with the study or safety of the individual.

### 2.2. Supplementation

The two trials consisted of four graded doses of cellulose-encapsulated phenylalanine or serine (one capsule, 0.5 g). Both encapsulated amino acids (Swanson Health Products, Fargo, ND, USA) were produced by Ajinomoto Health and Nutrition Inc., Raleigh, NC, USA. The starting dose (3.0 g/day) was comparable to the daily intake of either phenylalanine or serine from a regular diet [1,2]. This initial supplemental dose was assumed to be safe since the NHANES III set the 95th percentile intakes of phenylalanine or serine at more than 7 g/day without noting significant adverse effects [1]. Subsequent doses were determined based on experience with other amino acids [14] as an integer multiple of the starting dose (6, 9, and 12 g/day). Participants continued to the higher doses following a 2-week wash-out period at the discretion of the investigator.

### 2.3. Study Intervention, Sample Collection and Analysis

Subjects received supplements for the whole 4-week test period and were instructed to consume them daily before 12:00 pm, consistent with the previous research [15]. At the end of each 4-week test period, the subjects visited the clinic for safety evaluation following overnight fasting (minimum 8 h). Laboratory measurements included body weight, blood pressure and pulse rate. All biochemical analyses were conducted by LSI Medience Co., Ltd. (Osaka, Japan) using routine clinical methodologies. Biochemical serum analyses included white and red blood cell counts measured by an automated flow-cytometry analyzer Sysmex XE-2100 (Sysmex Co., Tokyo, Japan), hemoglobin, hematocrit and platelet count measured by red blood cell pulse peak detection method and the electrical resistance detection method, respectively (Sysmex XE-2100, Sysmex Co., Tokyo, Japan). Standard blood chemistry (glutamic oxaloacetic transaminase, glutamic pyruvic transaminase, gamma glutamyltransferase, alkaline phosphatase, lactate dehydrogenase, total bilirubin, creatinine, blood urea nitrogen, triglycerides, glucose, uric acid, creatine phosphokinase and total protein) was evaluated based on the methodologies recommended by the Japanese Society of Clinical Chemistry using Hitachi automated analyzer Labospect LST008α (Hitachi Co., Tokyo, Japan) combined with JCA-BM8000 automatic analyzer (JEOL Co., Inc., Tokyo, Japan).

Albumin was analyzed by dye binding-bromocresol purple method using Hitachi automated analyzed Labospect LST008α (Hitachi Co., Tokyo, Japan). Serum levels of electrolytes (sodium, potassium, chloride and calcium) were analyzed by the electrode method using Hitachi automated analyzer Labospect LST008α (Hitachi Co., Tokyo, Japan). Blood glucose was measured enzymatically using the JCA-BM9130 automatic analyzer (JEOL Co., Inc., Tokyo, Japan).

Plasma concentrations of free amino acids were measured by pre-column derivatization high-performance liquid chromatography (Agilent Co., Santa Clara, CA, USA) and homocysteine levels by liquid chromatography combined with mass spectrometry (Agilent Co., Santa Clara, CA, USA).

Compliance (intake of the tested amino acids) was assessed daily through an online survey. Another survey was used to measure relative changes in subjective sleep quality and mental fatigue using a linear scale ranging from −5 to +5, with zero corresponding to “no change” from the previous day, negative values indicating worsening, and positive values indicating improving. In addition, a dietary survey using the Sasaki Food Habit Assessment [16] was conducted at the end of each 4-week test period to assess the food intake status in terms of total food energy and main macronutrient categories (proteins, lipids, and carbohydrates). An individual adverse event was defined as an independent event when its endpoint was confirmed by the investigator. Low back pain, joint pain, gastrointestinal events, tiredness, headache and “cold-like” symptoms were considered adverse effects. Adverse events were assessed as unrelated, probably related, or related to the test compound by the investigator at the end of each 4-week test period.

### 2.4. Statistical Analyses

The statistical analyses were performed on the per-protocol set. In the phenylalanine trial, additional statistical analyses were also performed on the full “intention to supplement” set. Significant differences were determined using one-factor repeated-measures ANOVA followed by Tukey’s multiple group comparison significance test (SPSS Ver. 26, Japan IBM Co. Ltd., Tokyo, Japan). The level of significance was determined at *p* < 0.05. All results are presented as means ± standard errors (SEMs).

## 3. Results

### 3.1. Subjects in the Per-Protocol Set

In the phenylalanine group, 4 of the initial 30 subjects were withdrawn at the discretion of the investigator after receiving requests by the subjects due to causes unrelated to the study (three subjects were withdrawn due to anxiety about the COVID-19 pandemic, and one subject was withdrawn due to family reasons). In addition, the investigator judged that three subjects (ID 13035, 13069 and 13093) had breached the consumption protocol for phenylalanine capsule intake. Therefore, 23 subjects in the phenylalanine group were evaluated in the per-protocol set. In the serine group, no subjects were withdrawn, and no subjects breached the protocol. Consequently, all 30 initial subjects were evaluated in the per-protocol set.

### 3.2. Blood Biochemistry

The results of biochemical analyses conducted at the end of each 4-week test period are shown in Table 1. Phenylalanine administration (*n* = 23) triggered a small but statistically significant increase in serum sodium (6 g/day) and chloride (all tested doses) when compared to the baseline values. These changes were within the reference ranges of the clinic and were not found to be toxicologically relevant. Similarly, serine administration triggered a small but significant increase of serum sodium (6 g/day) and chloride (3–9 g/day), but all observed changes were within the reference ranges of the clinic and were not determined to be toxicologically relevant. In the phenylalanine trial, blood chemistry analyses were also repeated on the full “intention to supplement” set (*n* = 30). Identically to the per-protocol set analyses, a small but statistically significant increase in serum sodium (6 g/day) and chloride (all tested doses) were recorded (data not shown). No other changes in blood parameters were observed (Table 1).

Neither phenylalanine nor serine administration had a significant effect on plasma essential amino acids (Table 2). Among non-essential amino acids, phenylalanine supplementation triggered minor but statistically significant variations in circulating glutamine and aspartic acid at mid-doses and an increase in tyrosine (12 g/day). The statistical analysis was also repeated on the full “intention to supplement” set (*n* = 30). Identically to the results in the per-protocol set analyses (*n* = 23), phenylalanine supplementation decreased slightly but significantly plasma glutamine and aspartic acid at mid-doses (data not shown).

Serine supplementation (9 and 12 g/day) significantly increased serine plasma levels and led to a minor variation of glutamine when the dosage 3 g/day was compared to the highest tested dose of 12 g/day (Table 2).

### 3.3. Adverse Effects

During the phenylalanine supplementation, 25 adverse events were identified in seven subjects. These included 11 events of gastrointestinal symptoms, 7 events of headache, 4 events of common cold-like symptoms and 3 events of tiredness. Most of the events occurred during the initially tested doses of phenylalanine (3 and 6 g/day). At the highest dose (12 g/kg), one event of gastrointestinal symptoms and two events of cold-like symptoms occurred.

Among all recorded events, 23 events were mild and required no treatment, and 2 were moderate and therefore included brief pharmacological treatment. In one subject (ID 13015), gastrointestinal symptoms included mild diarrhea lasting 5 days (phenylalanine dose, 3 g/day) and another period of mild diarrhea lasting 2 days (phenylalanine dose, 6 g/day). However, the symptoms were judged unrelated to phenylalanine administration, and diarrhea was not reported in the subject at higher doses of phenylalanine (phenylalanine doses, 9 and 12 g/day). Causal relationships between the observed adverse events and phenylalanine administration were ruled out by the investigator.

During serine supplementation, four adverse events from three subjects were identified at the lowest tested dose (3 g/day). These included two events of gastrointestinal symptoms and two events of headache; among those, two events were mild in severity, and two events were moderate and, therefore, included short-lasting pharmacological treatment. No adverse events were identified at higher tested doses of serine (6, 9 and 12 g/day). Taken together, causal relationships between the observed adverse events and serine administration were ruled out by the investigator.

### 3.4. Sleep Quality, Mental Fatigue, Macronutrient and Caloric Intakes, Body Weight Changes

Daily online surveys of subjective sleep quality and mental fatigue were conducted daily from 7 days prior to the start of the intake of the tested amino acids until the end of the test period. The survey rated changes from the previous day for both fatigue and sleep quality using 11 subjective levels, with the worst condition being set as −5, no change being set as 0, and the best condition being set as +5. No significant effects of phenylalanine or serine supplementation on either mental fatigue or sleep quality were found (Table 3).

A food intake survey was conducted at the end of each 4-week period using the Sasaki-style food habit assessment [16], which was previously validated for Japanese subjects. No significant effects of either phenylalanine or serine on intake of total energy (kcal/day), protein (g/day), fat (g/day) and carbohydrates (g/day) were found (data not shown). No phenylalanine or serine administration-related changes in body weight were found (data not shown).

## 4. Discussion

To the best of our knowledge, these are the first dedicated dose-response clinical safety studies of either phenylalanine or serine. The results of the two trials indicate the subchronic clinical NOAEL for phenylalanine and serine at the highest dose tested (12 g/day).

In the case of phenylalanine, this conclusion correlates with observations made in previous efficacy studies of phenylalanine as a nutrient in specific subpopulations [17,18]. It is worth noticing that aspartame, a high-intensity sweetener, is manufactured by the coupling of esterified phenylalanine and aspartic acid and that phenylalanine has been included in previous reviews of aspartame safety [19]. However, aspartame consumption even at the 99th percentile of intake corresponds to the consumption of phenylalanine at approximately 1 g/day [20], which is substantially below the doses evaluated in this report. In the case of serine, peer-reviewed supplementation studies that would aid in discussing the current results within a broader perspective are missing. This may soon change due to recent findings of decreased serine levels in both the blood circulation and skeletal muscle with aging and disease [7].

While human safety data are limited, subchronic rodent toxicological studies of both phenylalanine and serine have already been published [21,22]. However, in the case of macronutrients such as amino acids, rodent findings have limited applicability to human risk assessment [23,24]. Macronutrients are not substances added to foods at milligram levels, such as newly developed flavors or artificial sweeteners that can indeed be studied in rodent tests at several hundred times their estimated human exposure. Macronutrients, including proteinogenic amino acids, have a long evolutionary history in foods and are ingested from regular food sources at doses of grams per day [1,2,3]. Feeding laboratory animals amino acids at amounts that are a hundred times higher than the regular dietary intake in order to use cross-species safety factors triggers non-specific and toxicologically irrelevant findings (e.g., a decrease in food intake) [23,24,25]. Therefore, animal data have limited relevance to human tolerance and interpretations of the current trials includes only human data.

Both tested amino acids slightly increased serum levels of chloride (from 102.4 to maximum 104.8 mmol/L (phenylalanine) and from 102.3 to maximum 104.7 mmol/L, (serine)). Because the effects were minor (within the normal range of 97–107 mmol/L), lacked a dose-dependent relationship, and, in the case of the phenylalanine trial, were identical in “per-protocol” and “intention to supplement” sets, the observations were not considered pathological.

Serine supplementation dose-dependently elevated serine plasma levels without affecting plasma levels of glycine or homocysteine. Homocysteine was specifically analyzed because serine is involved in homocysteine metabolism, and it was postulated that increased amounts of circulating serine might contribute to lowering plasma homocysteine [26]. Nevertheless, in the framework of this study, no changes in plasma homocysteine levels were found, even at the highest tested doses of serine (9 and 12 g/day). Circulating glycine, as such, has been proposed to be a biomarker of insulin resistance [27] or non-alcoholic fatty liver disease [28]. However, the lack of change in glycine concentrations respective to serine administration in this study suggests that dietary serine does not interfere with its potential use as a biomarker of disease or that the relationship is limited only to specific subgroups [28]. Finally, serine can also be diverted to its enantiomer (d-serine). Yet, because serine racemase, which catalyzes the stereochemical inversion of serine to d-serine, is present mainly in the brain [29], we hypothesized that serine supplementation would not have resulted in an increased circulating d-serine, and we did not evaluate it.

Phenylalanine is an essential amino acid rapidly converted (hydroxylated) to tyrosine. Thus, unsurprisingly [30], at the highest supplementation dosage, phenylalanine elevated levels of circulating tyrosine. On the other hand, as already mentioned, serine dose-dependently increased its plasma levels without being converted to glycine. That observation was similar to findings from a single female patient with an inherited metabolic disorder [31] and from patients with non-alcoholic fatty liver disease [32], who were also characterized by a serine supplementation-induced drop in circulating branched-chain amino acids, which was not seen in the present trial. In parallel to previous phenylalanine and serine studies [30,31,32] and to clinical safety studies with other amino acids [15,33], plasma changes in tyrosine and serine were not considered pathological because they were transient, unaccompanied by imbalances of other amino acids and unconnected to any changes in the evaluated behavioral parameters. An increase in plasma tyrosine measured when phenylalanine was supplemented at 12 g/day might have hypothetically affected the brain catecholamines and thus reduced stress and mental fatigue [34]. Yet, a survey of mental fatigue and sleep quality did not show any effects of the supplemented amino acids indicating that the increase in the plasma tyrosine level was insufficient to affect the measured mental parameters. Finally, despite a lack of pathological consequences, it cannot be excluded that the plasma changes of tyrosine or serine might have caused transient metabolic adaptations, but the presented methodology did not allow the detection of any.

In terms of other amino acids, both supplementations were associated with only marginal changes in plasma levels of glutamine and aspartic acid at mid-dosages (3 and 9 g/day). Those changes had neither a dose-dependent character nor a pathological significance and might have been non-specific outcomes due to chance alone, given the multiple comparisons made. This was also signified by the fact that the findings were also observed in the “intention to supplement” set (phenylalanine trial). Finally, it is unfeasible that the observed findings were due to cellulose capsules as such because of a lack of metabolic rationale or dose-dependency.

A range of mild adverse effects, most of them gastrointestinal, were described by the subjects in both the phenylalanine and serine groups. Only one patient reported minor bouts of diarrhea at low test doses of phenylalanine. Due to the fact that the tests were conducted without placebo controls, it was impossible to discriminate the non-specific gastrointestinal effects of ingesting cellulose capsules from the effects of the tested amino acids. Non-gastrointestinal adverse effects (headache, tiredness and cold-like symptoms) were mild, infrequent and happened mostly in the initial phases of the trial. Therefore, they were not considered to be causally linked to the supplemented amino acids.

The duration of supplementation (4 weeks) was chosen based on previous clinical studies [14,15,33] as both the most appropriate and practical period to evaluate tolerance to dietary intake of amino acids. On the other hand, this methodology did not allow quantifying the acute effects on blood biochemistry. Among others, the ingestion of phenylalanine (10 g/person) was reported to reduce postprandial glucose up to 2.5 h post-ingestion [35]. However, there were no effects of phenylalanine on blood glucose levels when evaluated in the present study at the end of 4-week test periods. This might be primarily related to the fact that in this subchronic trial, blood analyses were done in the postabsorptive state at least 24 h after the last amino acid dosing.

There are other limitations of the present research. First, while both supplemented amino acids had no discernible effects on body weight and food intake evaluated through online survey methods (including intake of major macronutrients), one cannot preclude that the supplementations affected intake of liquids and minor constituents of the daily diet, such as minerals. Second, no attempt was made to test doses higher than 12 g/day, and no clear marker of toxicity was found. The decision not to increase the dose further was based on previous experience with amino acids [33], as well as ethical concerns with requesting the subjects to ingest more than 30 capsules daily for a prolonged time, and practical reflections on current nutritional uses of both amino acids, which tend to be at 1–3 g/day (4). Third, the study was limited to male subjects of a single ethnic group.

In conclusion, the present results obtained in healthy male adults allow the authors to propose the highest tested dose (12 g/day) as a subchronic clinical NOAEL for both supplemental phenylalanine and supplemental serine.

## Figures and Tables

**Table 1:** Changes in biochemical blood parameters throughout the oral phenylalanine or serine supplementation period in healthy adults.

Blood Parameter	Trial	Phenylalanine (*n* = 23)	Serine (*n* = 30)
Total bilirubin (mg/L) Ref. value: 2–12	Baseline	9.1 ± 0.5	9.1 ± 0.6
	3 g/day	9.7 ± 0.6	9.9 ± 0.7
	6 g/day	7.4 ± 0.5	8.9 ± 0.5
	9 g/day	8.7 ± 0.7	9.6 ± 0.7
	12 g/day	8.0 ± 0.5	9.1 ± 0.7
Glutamic oxaloacetic transaminase (U/L) Ref. value: 10–40	Baseline	19.7 ± 0.8	21.0 ± 0.9
	3 g/day	18.3 ± 1.0	19.7 ± 1.3
	6 g/day	18.8 ± 0.7	19.2 ± 0.7
	9 g/day	20.9 ± 2.1	20.2 ± 1.1
	12 g/day	20.0 ± 1.2	20.4 ± 0.9
Glutamic pyruvic transaminase (U/L) Ref. value: 5–45	Baseline	18.7 ± 0.8	23.2 ± 2.0
	3 g/day	17.6 ± 2.0	19.8 ± 1.9
	6 g/day	17.7 ± 1.7	18.2 ± 1.6
	9 g/day	18.2 ± 1.7	19.3 ± 1.9
	12 g/day	18.7 ± 1.7	18.6 ± 1.8
Alkaline phosphatase (U/L) Ref. value: 100–325	Baseline	191.2 ± 9.8	230.1 ± 11.4
	3 g/day	185.1 ± 8.8	214.8 ± 0.2
	6 g/day	191.4 ± 10.1	218.4 ± 10.1
	9 g/day	186.8 ± 9.0	186.8 ± 9.0
	12 g/day	191.7 ± 9.0	191.7 ± 9.0
Lactate dehydrogenase (U/L) Ref. value: 120–240	Baseline	171.4 ± 4.7	162.7 ± 3.4
	3 g/day	178.1 ± 4.7	176.8 ± 7.0
	6 g/day	182.2 ± 4.2	174.0 ± 3.7
	9 g/day	190.1 ± 8.7	178.7 ± 4.1
	12 g/day	181.3 ± 4.9	173.0 ± 3.1
Gamma-glutamyltransferase (U/L) Ref. value: ≤80	Baseline	20.9 ± 2.1	29.1 ± 3.6
	3 g/day	21.6 ± 2.7	27.5 ± 3.0
	6 g/day	23.2 ± 2.7	26.0 ± 2.0
	9 g/day	22.8 ± 2.9	29.0 ± 3.8
	12 g/day	21.5 ± 2.5	30.3 ± 3.8
Creatine kinase (U/L) Ref. value: 60–270	Baseline	139.8 ± 12.8	121.4 ± 8.2
	3 g/day	149.4 ± 18.2	138.1 ± 12.9
	6 g/day	135.8 ± 13.4	135.4 ± 10.9
	9 g/day	318.8 ± 169.0	145.0 ± 5.9
	12 g/day	183.7 ± 33.6	158.8 ± 17.6
Total protein (g/L) Ref. value: 67–83	Baseline	72.8 ± 0.6	73.0 ± 0.7
	3 g/day	71.1 ± 0.7	71.4 ± 0.6
	6 g/day	70.6 ± 0.6	71.6 ± 0.6
	9 g/day	70.7 ± 0.6	71.0 ± 0.7
	12 g/day	71.3 ± 0.8	71.5 ± 0.6
Creatine (mg/L) Ref. value: 6.1–10.4	Baseline	8.6 ± 0.2	8.6 ± 0.2
	3 g/day	8.5 ± 0.2	8.4 ± 0.2
	6 g/day	8.4 ± 0.2	8.8 ± 0.2
	9 g/day	8.3 ± 0.2	8.4 ± 0.2
	12 g/day	8.5 ± 0.2	8.3 ± 0.2
Blood urea nitrogen (mg/L) Ref. value: 80–200	Baseline	130.3 ± 6.0	144.1 ± 6.3
	3 g/day	124.9 ± 5.5	139.4 ± 7.4
	6 g/day	133.4 ± 6.5	144.8 ± 6.3
	9 g/day	133.3 ± 6.9	143.3 ± 7.3
	12 g/day	135.7 ± 5.2	143.8 ± 5.9
Uric acid (mg/L) Ref. value: 38–70	Baseline	54.2 ± 2.2	61.0 ± 1.9
	3 g/day	55.7 ± 2.1	59.7 ± 2.0
	6 g/day	54.6 ± 1.9	61.9 ± 1.9
	9 g/day	58.6 ± 2.5	61.1 ± 2.0
	12 g/day	56.7 ± 1.8	59.9 ± 1.9
Total cholesterol (mg/L) Ref. value: 2100–2190	Baseline	1969 ± 74	2023 ± 47
	3 g/day	1860 ± 73	1913 ± 53
	6 g/day	1812 ± 68	1885 ± 54
	9 g/day	1837 ± 71	1906 ± 56
	12 g/day	1900 ± 59	1950 ± 57
Triglycerides (mg/dL) Ref. value: less than 147	Baseline	86.9 ± 9.2	96.7 ± 8.2
	3 g/day	75.4 ± 7.8	97.0 ± 9.3
	6 g/day	93.1 ± 17.8	90.4 ± 6.3
	9 g/day	79.1 ± 10.1	116.1 ± 12.1
	12 g/day	78.0 ± 7.8	100.9 ± 11.4
Sodium (mmol/L) Ref. value: 137–147	Baseline	140.3 ± 0.3	140.6 ± 0.2
	3 g/day	140.8 ± 0.3	140.4 ± 0.3
	6 g/day	141.6 ± 0.3 *	141.6 ± 0.4 ^$^
	9 g/day	141.3 ± 0.4	140.7 ± 0.3
	12 g/day	140.8 ± 0.2	140.6 ± 0.3
Potassium (mmol/L) Ref. value: 3.5–5.0	Baseline	4.27 ± 0.04	4.17 ± 0.05
	3 g/day	4.12 ± 0.05	4.06 ± 0.03
	6 g/day	4.17 ± 0.04	4.10 ± 0.04
	9 g/day	4.09 ± 0.05	4.02 ± 0.05
	12 g/day	4.24 ± 0.05	4.08 ± 0.04
Chloride (mmol/L) Ref. value: 97–107	Baseline	102.4 ± 0.3	102.4 ± 0.3
	3 g/day	104.4 ± 0.3 *	104.3 ± 0.4 *
	6 g/day	104.8 ± 0.3 *	104.7 ± 0.4 *
	9 g/day	104.8 ± 0.4 *	103.9 ± 0.3 *
	12 g/day	103.9 ± 0.3 *	103.3 ± 0.3
Calcium (mEq/L) Ref. value: 8.4–10.4	Baseline	9.24 ± 0.07	9.24 ± 0.05
	3 g/day	9.29 ± 0.07	9.29 ± 0.05
	6 g/day	9.26 ± 0.05	9.23 ± 0.05
	9 g/day	9.24 ± 0.04	9.25 ± 0.05
	12 g/day	9.37 ± 0.06	9.36 ± 0.06
HDL cholesterol (mg/dL) Ref. value: 40–85	Baseline	63.7 ± 2.3	61.7 ± 2.9
	3 g/day	61.7 ± 2.0	57.0 ± 3.0
	6 g/day	60.7 ± 2.1	58.8 ± 2.8
	9 g/day	60.8 ± 1.9	58.0 ± 3.0
	12 g/day	61.0 ± 1.7	61.3 ± 3.4
LDL cholesterol (mg/dL) Ref. value: 65–139	Baseline	116.2 ± 6.1	123.5 ± 4.8
	3 g/day	106.3 ± 5.8	112.9 ± 4.6
	6 g/day	101.4 ± 5.5	110.0 ± 4.9
	9 g/day	107.6 ± 5.9	111.6 ± 5.1
	12 g/day	112.2 ± 5.1	116.3 ± 5.0
Albumin (g/L) Ref. value: 38–52	Baseline	46.3 ± 0.7	46.5 ± 0.4
	3 g/day	45.4 ± 0.5	45.4 ± 0.4
	6 g/day	44.8 ± 0.6	45.7 ± 0.4
	9 g/day	45.0 ± 0.6	45.2 ± 0.3
	12 g/day	45.7 ± 0.7	45.9 ± 0.4
White blood cells (/mL) Ref. value: 3300–9900	Baseline	5287 ± 201	5400 ± 223
	3 g/day	5465 ± 244	5603 ± 221
	6 g/day	5961 ± 274	5490 ± 208
	9 g/day	5661 ± 241	5573 ± 255
	12 g/day	5813 ± 386	5493 ± 196
Red blood cells (×10^4^/mL) Ref. value: 430–580	Baseline	509.2 ± 5.4	507.2 ± 5.6
	3 g/day	502.6 ± 5.3	492.0 ± 5.1
	6 g/day	497.3 ± 6.5	494.4 ± 5.3
	9 g/day	493.5 ± 5.2	493.2 ± 5.2
	12 g/day	503.0 ± 6.9	497.4 ± 5.1
Hemoglobin (g/dL) Ref. value: 13.5–17.5	Baseline	15.86 ± 0.21	15.55 ± 0.21
	3 g/day	15.65 ± 0.21	15.07 ± 0.17
	6 g/day	15.47 ± 0.23	15.18 ± 0.20
	9 g/day	15.35 ± 0.22	15.10 ± 0.20
	12 g/day	15.66 ± 0.26	15.23 ± 0.16
Hematocrit (%) Ref. value: 39.7–52.4	Baseline	47.54 ± 0.50	46.78 ± 0.50
	3 g/day	48.17 ± 0.52	46.64 ± 0.45
	6 g/day	48.23 ± 0.69	47.14 ± 0.54
	9 g/day	47.80 ± 0.55	47.05 ± 0.53
	12 g/day	48.17 ± 0.58	46.86 ± 0.41
Platelet (×10^4^/mL) Ref. value: 14–34	Baseline	27.02 ± 0.84	26.09 ± 0.75
	3 g/day	26.30 ± 0.85	26.01 ± 0.81
	6 g/day	26.12 ± 0.79	25.37 ± 0.86
	9 g/day	26.14 ± 0.78	25.52 ± 0.77
	12 g/day	26.60 ± 0.87	25.10 ± 0.70
Glucose (mg/dL) Ref. value: 70–109	Baseline	88.2 ± 1.5	87.1 ± 1.4
	3 g/day	90.0 ± 1.5	87.5 ± 1.0
	6 g/day	90.1 ± 1.5	92.2 ± 1.9
	9 g/day	87.1 ± 1.6	91.7 ± 1.4
	12 g/day	87.1 ± 1.3	89.7 ± 2.0

Values represent mean ± SEM. One-way ANOVA was followed with Tukey’s multiple comparisons test between each amino acid intake: * *p* < 0.05 from baseline, ^$^ *p* < 0.05 from dosage of 3 g/kg.

**Table 2:** Changes in plasma free amino acid levels throughout the oral phenylalanine or serine supplementation period in healthy adults.

Essential Amino Acids (mmol/L)	Trial	Phenylalanine (*n* = 23)	Serine (*n* = 30)
Histidine	Baseline	84.0 ± 1.6	86.5 ± 1.4
	3 g/day	85.1 ± 1.6	84.5 ± 1.9
	6 g/day	83.8 ± 1.4	82.5 ± 1.6
	9 g/day	82.0 ± 1.1	82.0 ± 1.6
	12 g/day	84.3 ± 1.7	83.3 ± 1.6
Isoleucine	Baseline	63.7 ± 2.1	74.6 ± 3.9
	3 g/day	66.7 ± 2.5	72.3 ± 3.9
	6 g/day	72.1 ± 5.0	71.4 ± 3.0
	9 g/day	66.3 ± 3.1	74.5 ± 4.5
	12 g/day	69.7 ± 2.7	75.4 ± 3.6
Leucine	Baseline	124.4 ± 3.8	138.8 ± 6.8
	3 g/day	127.4 ± 3.6	137.6 ± 6.9
	6 g/day	133.6 ± 5.8	131.7 ± 3.9
	9 g/day	124.4 ± 4.9	137.1 ± 7.2
	12 g/day	131.1 ± 4.3	140.3 ± 5.5
Lysine	Baseline	197.3 ± 4.5	203.7 ± 8.4
	3 g/day	201.1 ± 5.1	193.8 ± 5.6
	6 g/day	194.6 ± 5.7	191.8 ± 4.9
	9 g/day	191.4 ± 5.7	196.5 ± 5.5
	12 g/day	207.4 ± 6.0	204.9 ± 6.5
Methionine	Baseline	28.3 ± 0.9	29.2 ± 1.1
	3 g/day	28.5 ± 0.8	27.7 ± 0.9
	6 g/day	28.7 ± 0.3	27.8 ± 0.7
	9 g/day	27.5 ± 1.1	28.2 ± 0.9
	12 g/day	28.1 ± 0.6	28.7 ± 1.1
Phenylalanine	Baseline	61.9 ± 1.7	62.2 ± 1.5
	3 g/day	65.1 ± 2.7	61.6 ± 1.5
	6 g/day	73.6 ± 5.2	60.9 ± 1.3
	9 g/day	68.6 ± 3.7	60.2 ± 1.8
	12 g/day	69.4 ± 3.8	59.3 ± 1.3
Threonine	Baseline	133.0 ± 4.4	138.8 ± 5.2
	3 g/day	131.3 ± 4.0	128.7 ± 4.7
	6 g/day	124.7 ± 5.0	131.2 ± 3.5
	9 g/day	123.8 ± 5.0	127.3 ± 3.8
	12 g/day	124.7 ± 3.7	135.2 ± 4.6
Tryptophan	Baseline	52.1 ± 1.2	59.3 ± 2.1
	3 g/day	53.9 ± 1.3	56.8 ± 2.7
	6 g/day	54.0 ± 1.4	57.4 ± 1.6
	9 g/day	52.2 ± 1.6	56.5 ± 2.1
	12 g/day	52.0 ± 1.3	54.7 ± 2.0
Valine	Baseline	237.0 ± 5.7	261.7 ± 10.1
	3 g/day	238.4 ± 5.9	254.5 ± 11.1
	6 g/day	249.0 ± 8.4	248.6 ± 7.9
	9 g/day	238.3 ± 8.0	252.5 ± 10.6
	12 g/day	251.8 ± 7.5	259.8 ± 8.3
**Non-Essential Amino Acids, Cystine and Homocysteine (mmol/L)**	**Trial**	**Phenylalanine (*n* = 23)**	**Serine (*n* = 30)**
Alanine	Baseline	379.8 ± 17.9	410.9 ± 14.8
	3 g/day	391.5 ± 15.6	384.2 ± 17.9
	6 g/day	384.8 ± 20.0	391.8 ± 17.3
	9 g/day	378.0 ± 18.2	396.4 ± 16.0
	12 g/day	381.8 ± 15.4	385.1 ± 16.5
Arginine	Baseline	98.0 ± 2.6	101.7 ± 3.3
	3 g/day	94.8 ± 3.1	92.8 ± 2.7
	6 g/day	88.8 ± 3.1	90.8 ± 2.7
	9 g/day	86.5 ± 2.7	93.2 ± 3.4
	12 g/day	91.2 ± 2.4	95.5 ± 3.6
Aspartic acid	Baseline	2.2 ± 0.2	2.8 ± 0.3
	3 g/day	1.5 ± 0.3	1.7 ± 0.2
	6 g/day	2.0 ± 0.2	2.1 ± 0.2
	9 g/day	2.4 ± 0.2 ^$^	2.3 ± 0.2
	12 g/day	1.6 ± 0.3	2.1 ± 0.3
Asparagine	Baseline	59.4 ± 1.3	61.1 ± 1.9
	3 g/day	61.2 ± 1.4	58.1 ± 1.8
	6 g/day	60 ± 2.3	59.2 ± 2.0
	9 g/day	58.3 ± 1.9	59.1 ± 1.9
	12 g/day	58.2 ± 1.4	57.4 ± 1.9
Cystine	Baseline	50.3 ± 1.5	51.2 ± 1.6
	3 g/day	49.0 ± 1.6	49.2 ± 1.5
	6 g/day	47.4 ± 1.5	48.1 ± 1.4
	9 g/day	48.5 ± 1.4	49.8 ± 1.6
	12 g/day	46.4 ± 1.4	47.9 ± 1.4
Glutamic acid	Baseline	32.4 ± 1.7	39.7 ± 2.2
	3 g/day	35.5 ± 2.2	37.0 ± 2.0
	6 g/day	36.5 ± 2.6	39.5 ± 1.8
	9 g/day	35.1 ± 2.4	40.3 ± 2.0
	12 g/day	28.2 ± 2.3	36.6 ± 2.5
Glutamine	Baseline	559.6 ± 10.5	527.4 ± 8.6
	3 g/day	547.3 ± 10.9	518.4 ± 9.6 ^#^
	6 g/day	522.5 ± 12.4	495.9 ± 9.5
	9 g/day	513.6 ± 9.4 *^,#^	502.2 ± 9.6
	12 g/day	559.4 ± 10.5	537.9 ± 10.2
Glycine	Baseline	244.6 ± 8.5	257.5 ± 12.4
	3 g/day	248.0 ± 8.5	250.0 ± 11.3
	6 g/day	230.2 ± 10.0	264.8 ± 13.6
	9 g/day	229.0 ± 9.1	259.7 ± 12.0
	12 g/day	228.0 ± 7.4	269.7 ± 13.0
Proline	Baseline	164.6 ± 6.8	186.6 ± 8.5
	3 g/day	162.1 ± 6.7	178.4 ± 9.2
	6 g/day	167.9 ± 10.0	182.3 ± 8.2
	9 g/day	157.3 ± 6.2	182.5 ± 11.0
	12 g/day	154.5 ± 5.6	178.2 ± 9.5
Serine	Baseline	113.9 ± 3.4	117.3 ± 4.0
	3 g/day	119.7 ± .9	122.5 ± 3.6
	6 g/day	113.0 ± 4.4	138.7 ± 5.6
	9 g/day	113.1 ± 3.5	157.1 ± 7.2 **
	12 g/day	113.9 ± 3.5	175.5 ± 12.7 **
Tyrosine	Baseline	62.3 ± 1.8	67.2 ± 2.1
	3 g/day	66.7 ± 2.2	62.9 ± 2.5
	6 g/day	72.4 ± 3.6	63.5 ± 2.1
	9 g/day	67.1 ± 3.7	61.6 ± 2.8
	12 g/day	75.0 ± 4.2 *	62.1 ± 2.4
Homocysteine	Baseline		12.6 ± 0.6
	3 g/day		12.8 ± 1.4
	6 g/day		13.8 ± 2.0
	9 g/day		13.9 ± 2.2
	12 g/day		13.4 ± 2.0

Values represent mean ± SEM. One-way ANOVA was followed with the Tukey’s multiple comparisons test between each amino acid intake: * *p* < 0.05, ** *p* < 0.01 from baseline, ^$^ *p* < 0.05 from dosage of 3 g/kg, ^#^ *p* < 0.05 from dosage of 12 g/kg.

**Table 3:** Self-evaluated changes in sleep quality and mental fatigue throughout the oral phenylalanine or serine supplementation period in healthy adults.

Sleep Quality	Phenylalanine (*n* = 23)	Serine (*n* = 30)
Baseline (7 days)	0.1801 ± 0.0769	0.3333 ± 0.1300
3 g/day (28 days)	0.2474 ± .0812	0.3058 ± 0.1442
Wash-out period (14 days)	0.1137 ± 0.1012	0.1667 ± 0.1271
6 g/day (28 days)	0.1352 ± 0.1069	0.2437 ± 0.1335
Wash-out period (14 days)	0.0648 ± 0.0718	0.1846 ± 0.1168
9 g/day (28 days)	0.0753 ± 0.0577	0.3171 ± 0.1815
Wash-out period (14 days)	0.0001 ± 0.0181	0.2949 ± 0.1840
12 g/day (28 days)	0.1316 ± 0.0615	0.3295 ± 0.1848
**Mental fatigue**	**Phenylalanine (*n* = 23)**	**Serine (*n* = 30)**
Baseline (7 days)	0.1180 ± 0.0513	0.2905 ± 0.1428
3 g/day (28 days)	0.1934 ± 0.0887	0.2908 ± 0.1310
Wash-out period (14 days)	0.1104 ± 0.1066	0.1692 ± 0.1236
6 g/day (28 days)	0.1098 ± 0.1007	0.2678 ± 0.1212
Wash-out period (14 days)	0.0715 ± 0.0657	0.1872 ± 0.1275
9 g/day (28 days)	0.0317 ± 0.0706	0.3194 ± 0.1829
Wash-out period (14 days)	0.0268 ± 0.0308	0.3026 ± 0.1800
12 g/day (28 days)	0.0946 ± 0.0457	0.3360 ± 0.1856

Values represent mean ± SEM of all tested subjects (n) over the whole period of supplementation (28 days), baseline (7 days) and wash-out recovery periods (14 days). One-way ANOVA was followed with Tukey’s multiple comparisons test between each amino acid intake, baseline and wash-out period.

## Data Availability

The data presented in this study are available on request from the corresponding author.
